# Transcatheter patent foramen ovale closure versus medical therapy for cryptogenic stroke: a meta-analysis of randomized clinical trials

**DOI:** 10.1186/1471-2261-13-116

**Published:** 2013-12-11

**Authors:** Irbaz Bin Riaz, Abhijeet Dhoble, Ahmad Mizyed, Chiu-Hsieh Hsu, Muhammad Husnain, Justin Z Lee, Kapildeo Lotun, Kwan S Lee

**Affiliations:** 1 Department of Internal Medicine, University of Arizona, Tucson, AZ 85714, USA; 2 Department of Cardiovascular Diseases, University of Arizona, 3950 S Country Club Road, Suite 200, Tucson, AZ 85714, USA; 3 Department of Biostatistics, University of Arizona, Tucson, AZ 85714, USA

**Keywords:** Patent foramen ovale, Inter atrial shunt, Transcatheter closure, Cryptogenic stroke

## Abstract

**Background:**

There is an association between cryptogenic stroke and patent foramen ovale (PFO). The optimal treatment strategy for secondary prevention remains unclear. The purpose of this study was to analyze aggregate data examining the safety and efficacy of transcatheter device closure versus standard medical therapy in patients with PFO and cryptogenic stroke.

**Methods:**

A search of published data identified 3 randomized clinical trials for inclusion. The primary outcome was a composite end-point of death, stroke and transient-ischemic attack (TIA). Pre-defined subgroup analysis was performed with respect to baseline characteristics including age, sex, atrial septal aneurysm and shunt size. Data was synthesized using a random effects model and results presented as hazard ratios (HRs) with 95% confidence intervals (CIs).

**Results:**

A cohort of 2,303 patients with a history of cryptogenic stroke and PFO were randomized to device closure (n = 1150) and medical therapy (n = 1153). Mean follow-up was 2.5 years. Transcatheter closure was not superior to medical therapy in the secondary prevention of stroke or TIA in intention-to-treat analysis (HR: 0.66, 95% CI: 0.43 to 1.01; p = 0.056). However, the results were statistically significant using per-protocol analysis (HR: 0.64, 95% CI: 0.41 to 0.98; p = 0.043). Males had significant benefit with device closure (HR: 0.48, 95% CI: 0.24 to 0.96; p = 0.038).

**Conclusions:**

In this meta-analysis, using intention-to-treat analysis, transcatheter device closure of PFO was not superior to standard medical therapy in the secondary prevention of cryptogenic stroke. Transcatheter closure was superior using per-protocol analysis.

## Background

It is believed that up to 30-40% of strokes are cryptogenic in nature [1-5]. Multiple observational studies have demonstrated an association between cryptogenic stroke and patent foramen ovale (PFO) [6-15]. The prevalence of PFO in the general community is around 25 to 30% of individuals based on autopsy and community based transesophageal echocardiography (TEE) studies [16,17]. The prevalence of PFO is about 60% in patients with cryptogenic strokes [18], supporting an etiological association. These associations suggest that paradoxical embolism may be the cause of stroke in some of these patients.

Most patients with cryptogenic stroke are less than 55 years of age with significant cost implications both in the short and long term [17,19-21]. The optimal treatment strategy of secondary prevention for patients with cryptogenic stroke is still unclear. To date, there have been several observational studies and three randomized trials evaluating the safety and efficacy of transcatheter closure versus medical therapy in reducing the risk of recurrent stroke in this patient population [22-24]. Two recent meta-analyses of observational studies favored transcatheter closure over medical therapy in preventing recurrent strokes [25,26]. However, observational studies are limited by methodology and selection bias, whereas randomized trials provide the best scientific evidence for minimizing these biases. In this study, we only assessed the totality of evidence from the three recently published randomized trials on this subject. The main objectives of our meta-analysis were: 1) pool the aggregate data from these trials, thereby increasing the sample size and possibly reducing type 2 error; 2) explore the possibility of any particular sub-group that may derive benefit from the closure device; and 3) to assess the safety and complication rates of the procedure.

## Methods

### Search strategy

We searched Medline (via Ovid SP and PubMed), EMBASE, CINAHL and Cochrane Central Register For Controlled Trials; for eligible studies with the terms “patent foramen ovale”, “PFO”, “heart septal defects (atrial)”, “inter-atrial shunt”, “atrial septal aneurysm”, “ASA”, ”Transcatheter closure”, “recurrent stroke”, “recurrent TIA”, “cryptogenic stroke” and “recurrent thromboembolism”. In addition, abstracts and conference proceedings were hand searched where available. Reference lists from each article were scanned for further review. The search and extraction was performed according to the PRISMA statement [27].

### Study characteristics

We searched all the databases for randomized controlled trials comparing medical therapy versus transcatheter closure of PFO for secondary prevention of cryptogenic stroke/TIA. In addition, a qualified reference librarian independently searched similar databases using the same search terms. Studies that met the eligibility criteria were included in the meta-analysis.

### Data collection

Two independent investigators reviewed all articles for inclusion, with any disagreement resolved by consensus. The reviewers independently extracted any variables that described the study population, intervention description, and outcome data. A structured template was used to extract the relevant data.

### Outcome measures

The primary outcome was a composite of recurrent stroke, TIA or death during the mean follow up period of 2.5 years. Sub-group analysis was performed with respect to age, sex, atrial septal aneurysm and shunt size, where reported.

### Statistics

Data from individual studies were pooled using a random effects model. Hazard Ratio (HR), reported with 95% confidence intervals (CI) was used as a measure of effect with HR < 1 favoring device closure. A p-value of at least 0.05 was defined as statistically significant for the observed difference. Statistical heterogeneity was measured using Cochran’s Q statistic with α = 0.05 as well as the I^2^ statistic which is a measure of the proportion of the total variability due to heterogeneity beyond chance. I^2^ values of greater than 50% are consistent with significant heterogeneity.

## Results

**Figure 1:**
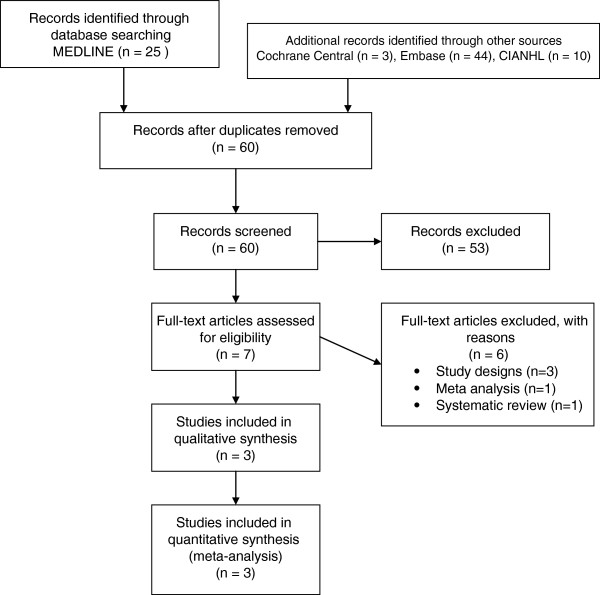
Flow diagram of trial selection process.

The results of the literature search are summarized in Figure 1. The COCHRANE search identified 3; Medline 25; EMBASE 44 and CINAHL 10 potentially eligible trials. Review of conference abstracts and reference lists did not identify any additional trials. Using a well-formulated search strategy (Additional file 1), a total of 60 records (after removing duplicates) were screened for inclusion in the meta-analysis. Three randomized controlled trials met our inclusion criteria and were included in the final analysis. The CLOSURE I Trial was a large, multi-center, randomized, clinical trial in which a total of 909 patients were enrolled between June 2003, and October 2008, utilizing the STARFlex device. The Amplatzer PFO closure device was studied in both the PC Trial, a multi-center, randomized trial performed in Europe, Canada, Brazil and Australia, enrolling 414 patients over 9 years; and RESPECT, a large, US multi-center trial enrolling 980 patients from August 2003 to December 2011. The results and methodological quality of the 3 trials are summarized in Table 1. All trials had excellent randomization procedures, and intention-to-treat analyses were reported for primary outcomes. Methods for allocation concealment were not clearly described. Although the adjudication of the end point was blinded, ascertainment of end points was probably un-blinded except in the RESPECT trial. There was no significant heterogeneity present amongst trials (I^2^ < 50).

**Table 1:** Summary of methodological assessment

	*Randomization Method Described	**Allocation concealment	**Blinding of participants, investigators, outcome assessors and data assessors**	***Intention to Treat Analysis	**Compliance Checked**
**CLOSURE**	Yes	Unclear but probably yes	Detailed information not clearly provided. Likely that ascertainment of endpoint was unblinded.	Yes	Yes
**RESPECT**	Yes	Unclear but probably yes	Detailed information not clearly provided. Likely that ascertainment of endpoint was unblinded.	Yes	Yes
**PC**	Yes	Unclear but probably yes	Outcome and Data Assessors were blinded.	Yes	Yes

*Randomization method if described appropriately was labeled as yes and no otherwise.

**Allocation concealment was categorized as adequate, unclear, and inadequate.

***Intention to treat was categorized as yes, unclear, no.

**Figure 2:**
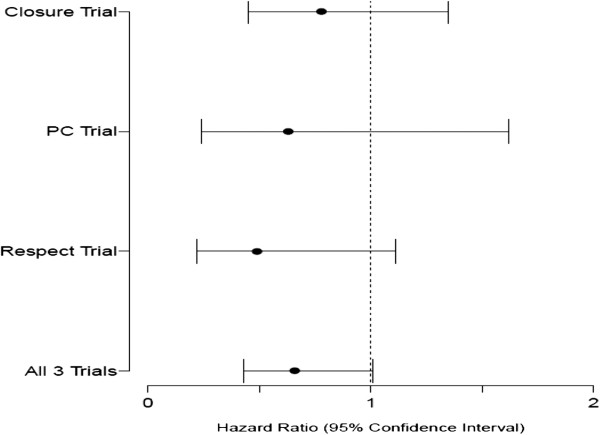
Forest plot showing intention-to-treat analysis of primary end point for all three randomised clinical trials.

**Figure 3:**
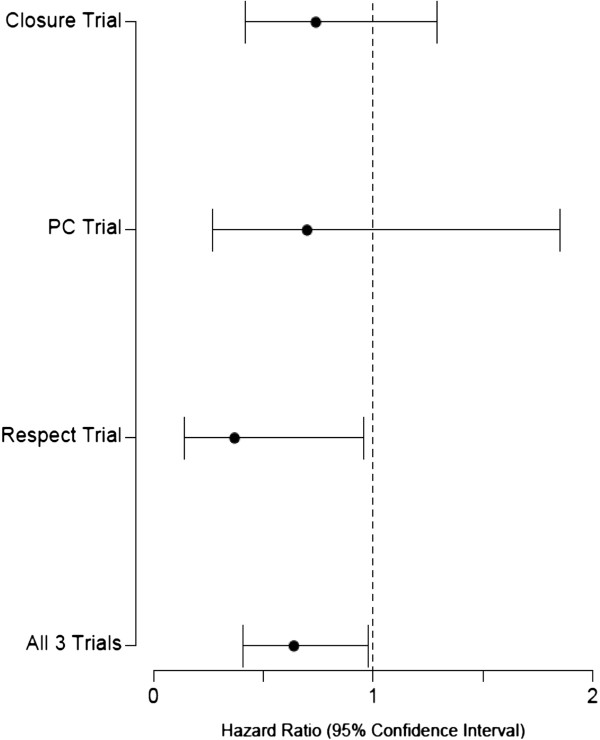
Forest Plot showing per-protocol analysis of primary end points for all three randomised clinical trials.

**Figure 4:**
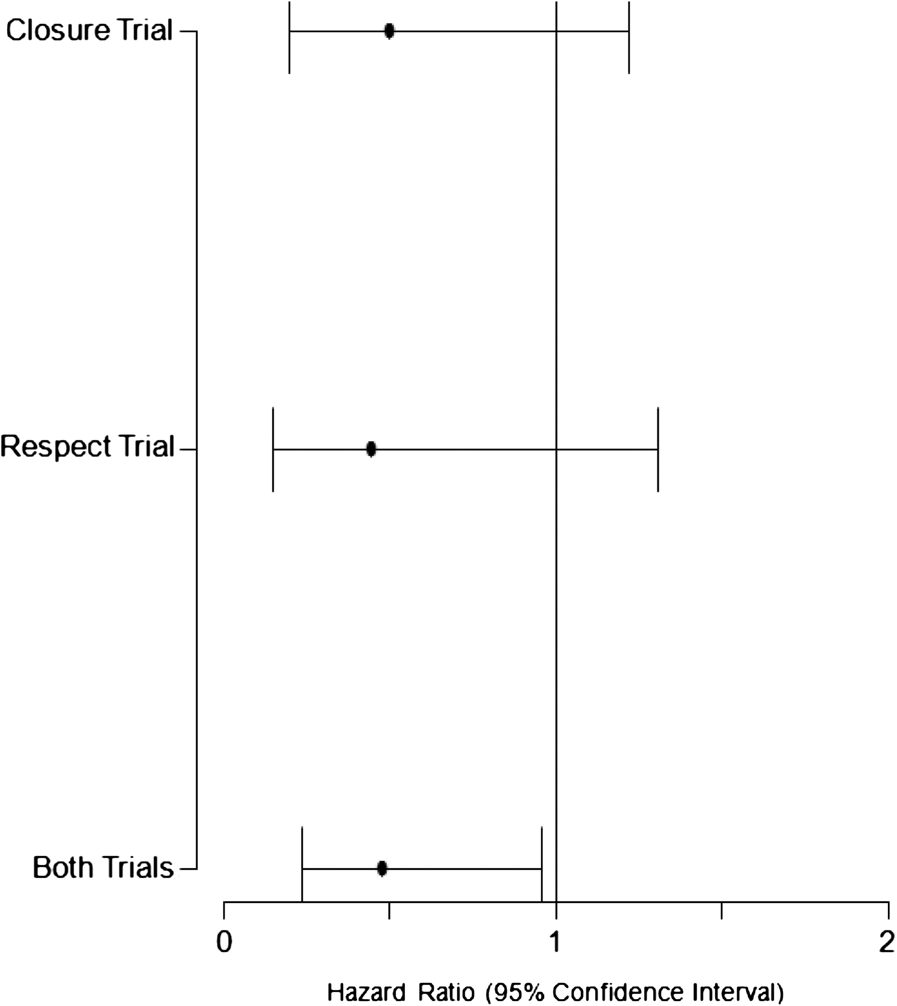
Forest plot showing subgroup analysis of primary end point for male population (CLOSURE and RESPECT Trial).

**Figure 5:**
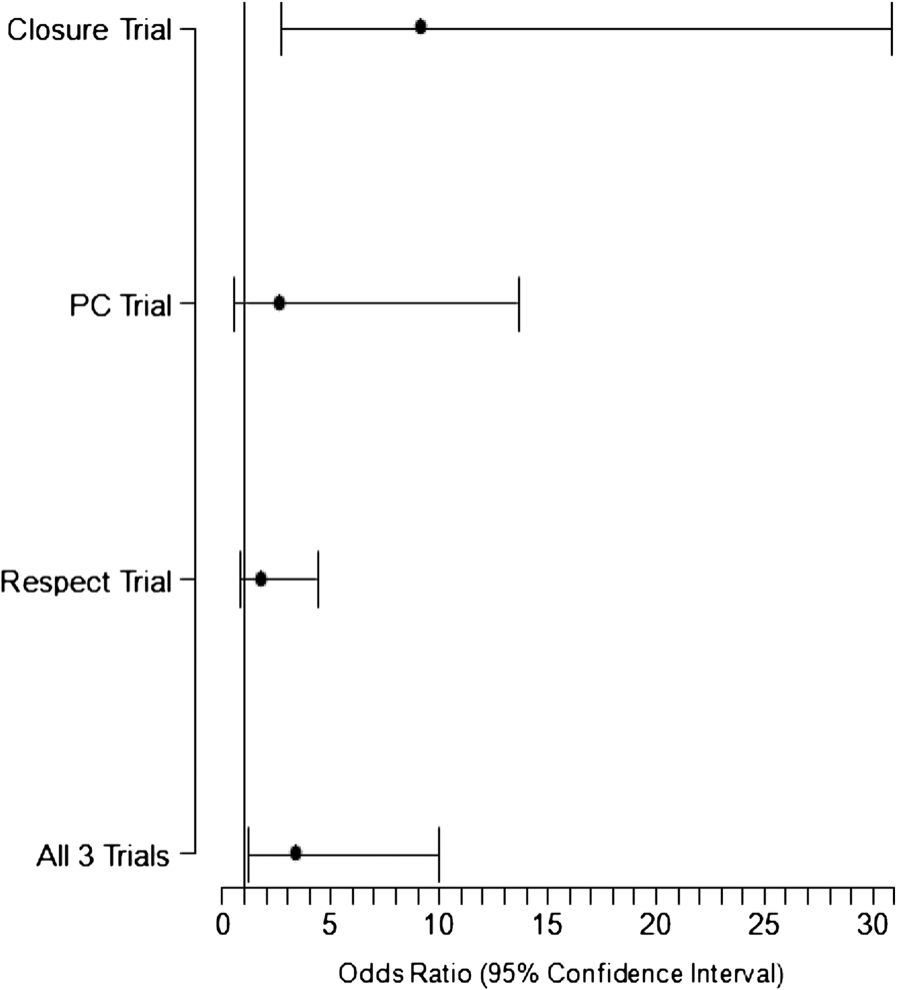
Forest plot showing analysis of atrial fibrillation in all three clinical trials.

A total of 2303 patients with a prior history of cryptogenic stroke were randomized; 1150 to device closure and 1153 to medical therapy. The study characteristics and baseline characteristics of the participants are summarized in Table 2. We found that transcatheter closure was not superior to standard medical treatment in the secondary prevention of strokes/TIA in patients with a PFO during a mean period of 2.5 years (maximum, 7 years) according to intention to treat analysis (HR 0.66, 95% CI 0.43-1.01, p = 0.056), with a trend favoring device closure. When analyzed using per-protocol method, transcatheter closure was superior to standard medical therapy in preventing recurrent events (HR: 0.64, 95% CI: 0.41-0.98; p = 0.043). Interestingly, we found that in the male population, reported in CLOSURE 1 and RESPECT, device closure was significantly associated with a reduction of recurrent events even with intention-to-treat analysis (HR 0.48, 95% CI 0.24-0.96, p = 0.038). No other subgroup clearly benefitted from device closure. Our analysis failed to confirm the benefit of transcatheter closure in patients with atrial septal aneurysm. The details of subgroup analysis are described in Table 3. The complication rates from transcatheter closure and medical treatment are summarized in Table 4. Major bleeding rates were similar in both groups, but atrial fibrillation occurred more often in the device group (HR 3.43, 95% CI 1.17-10.00, p = 0.024). The meta-analysis and forest plots are shown in Tables 5, 6 and 7 and Figures 2, 3,4 and 5 respectively.

**Table 2:** Study characteristics and baseline characteristics of participants

	**CLOSURE**		**PC**		**RESPECT**	
**Study Characteristics**						
**Design**	Randomized Controlled trial		Randomized Controlled trial		Randomized Controlled trial	
**Duration of follow up**	2.0 years		4.1 years		2.1 years	
**Location**	Multi-center trial (North America)		Multi-center trial (Europe, Canada, Brazil, Australia)		Multi-center trial (North America)	
**Total**	909		414		980	
	Closure	Medical	Closure	Medical	Closure	Medical
	447	462	204	210	499	481
**Participants**						
**Age**	46.3+/−9.6	45.7+/−9.1	44.3+/−10.2	44.6+/−10.1	45.7+/−9.7	46.2+/−10.0
**Male sex**	52.1%	51.5%	45.1%	54.3%	53.7%	55.7%
**Race or ethnic group**						
**Asian**	1.6%	1.7%	NA	NA	NA	NA
**Black**	4.2%	5.6%	NA	NA	NA	NA
**White**	89.0%	89.6%	NA	NA	NA	NA
**Hispanic**	6.7%	4.8%	NA	NA	NA	NA
**Smoking during previous year**	21.5%	22.6%	22.5%	22.4%	15.0%	11.4%
**Birth control/HRT**	NA	NA	NA	NA	8.2%	10.8%
**Deep venous thrombosis**	NA	NA	NA	NA	4.0%	3.1%
**Medical history (%)**						
**Hypertension**	33.8%	28.4%	24.0%	27.6%	31.7%	31.2%
**Hypercholesterolemia**	47.4%	40.9%	24.5%	29.5%	38.9%	40.1%
**Diabetes Mellitus**	NA	NA	2.5%	2.9%	6.6%	8.3%
**Migraine**	NA	NA	23%	18.1%	39.1%	38.5%
**Family history of cardiovascular disease or cerebrovascular accidents**	55.3%	55.6%	26.0%	19.0%	27.3%	22.5%
**Congestive Heart Failure**	0.4%	0%	NA	NA	0.6%	0%
**Ischemic heart disease**	1.3%	0.9%	2.0%	1.9%	3.8%	1.9%
**Myocardial infarction**	1.6%	1.1%	1.5%	0.5%	1.0%	0.4%
**Valvular dysfunction**	11.0%	9.7%	3.9%	2.4%	NA	NA
**Arrhythmia**	5.8%	4.1%	NA	NA	NA	NA
**Peripheral vascular disease**	1.1%	1.5%	1.5%	1.0%	1.0%	0.2%
**Pulmonary embolus**	0	0.9%	NA	NA	NA	NA
**Peripheral embolism**	NA	NA	2.9%	2.4%	NA	NA
**Previous TIA**	NA	NA	NA	NA	11.6%	12.7%
**Previous Stroke**	NA	NA	NA	NA	10.6%	10.6%
**Index neurologic event for study entry**						
**Stroke**	NA	NA	80.9%	77.6%	NA	NA
**Cryptogenic stroke**	72.6%	71.4%	NA	NA	100.0%	100.0%
**TIA**	27.4%	28.6%	16.2%	20.0%	NA	NA

**Table 3:** Summary of subgroup analysis

**Subgroup**	**CLOSURE Trial**		**PC Trial**		**RESPECT Trial**	
	**Closure**	**Medical**	**Closure**	**Medical**	**Closure**	**Medical**
**Sex (%)**						
**Male**	3.4	6.8	NA	NA	1.9	3.7
**Female**	7.9	7.0	NA	NA	1.7	2.8
**Atrial Septal Aneurysm (%)**						
**No**	6.2	7.4	1.9	5.7	2.2	2.2
**Yes**	4.6	6.0	8.5	3.9	1.1	5.3
**Age (%)**						
**≤ 45 yr**	NA	NA	1.1	6.2	1.7	2.4
**> 45 yr**	NA	NA	5.3	4.4	1.9	4.1
**Cardiovascular index event (%)**						
**Stroke**	5.1	5.1	3.0	4.9	NA	NA
**TIA or Peripheral Embolism**	7.1	11.6	5.1	6.4	NA	NA
**Shunt size (%)**						
**None to Moderate**	5.7	6.9	NA	NA	2.8	2.5
**Substantial**	3.5	4.9	NA	NA	0.8	4.3

**Table 4:** Summary of adverse events/complications

**Event**	**CLOSURE Trial**		**PC Trial**		**RESPECT Trial**	
	**Closure**	**Medical therapy**	**Closure**	**Medical therapy**	**Closure**	**Medical therapy**
**Major vascular procedural complication (%)**	3.2	NA	1.5	NA	0.6	NA
**Atrial fibrillation (%)**	5.7	0.7	2.9	1.0	3.0	1.5 %
**Major bleeding episode (%)**	2.6	1.1	3.9	5.7	1.6	1.9
**Death other than end point (%)**	0.5	0.9	NA	NA	NA	NA
**Nervous system disorder (%)**	1.5	3.5	NA	NA	NA	NA
**PFO-related hospital admission (%)**	NA	NA	6.4	6.2	NA	NA
**Dizziness (%)**	NA	NA	0.5	1.9	NA	NA
**Seizure (%)**	NA	NA	0.5	1.4	NA	NA
**Dyspnea (%)**	NA	NA	0	1.9	NA	NA
**Chest pain (%)**	NA	NA	1.5	1.9	NA	NA
**Allergic drug reaction (%)**	NA	NA	0.5	1.0	0.2	NA

**Table 5:** Primary endpoint meta-analysis

**Endpoint**	**Closure Trial**		**PC Trial**		**Respect Trial**		**Random Effects Model**		
	**HR**	**95%CI**	**HR**	**95%CI**	**HR**	**95%CI**	**HR**	**95% CI**	**p-value**
**ITT Population**									
Composite endpoint of all devices	0.78	0.45,1.35	0.63	0.24,1.62	0.49	0.22,1.11	0.66	0.43,1.01	0.06
Composite endpoint of Amplatzer device			0.63	0.24,1.62	0.49	0.22,1.11	0.54	0.29,1.01	0.05
**Per Protocol**									
Composite endpoint of all devices	0.74	0.42,1.29	0.70	0.27,1.85	0.37	0.14,0.96	0.64	0.41,0.98	0.04
Composite endpoint of Amplatzer device			0.70	0.27,1.85	0.37	0.14,0.96	0.64	0.44,0.97	0.03

**Table 6:** Subgroup analysis of the primary endpoint

**Subgroup**	**Closure Trial**		**PC Trial**		**Respect Trial**		**Random Effects Model**		
	**HR**	**95%CI**	**HR**	**95%CI**	**HR**	**95%CI**	**HR**	**95% CI**	**p-value**
**Atrial Septal Aneurysm**									
**Yes**	0.78	0.30, 2.13	2.09	0.38, 11.4	0.19	0.04, 0.87	0.67	0.21, 2.16	0.50
**No**	0.81	0.42, 1.59	0.32	0.09, 1.18	0.89	0.31, 2.54	0.71	0.43, 1.19	0.19
**Age**									
≤ 45	NA	NA	0.16	0.02, 1.31	0.70	0.19, 2.60	0.42	0.11, 1.66	0.22
> 45	NA	NA	1.22	0.37, 3.99	0.41	0.14, 1.17	0.68	0.23, 2.00	0.48
**Sex**									
**Male**	0.50	0.20, 1.22	NA	NA	0.45	0.15, 1.31	0.48	0.24, 0.96	0.04
**Female**	1.13	0.55, 2.34	NA	NA	0.57	0.16, 2.02	0.96	0.51, 1.79	0.89
**Shunt Size**									
**None, trace or moderate**	0.78	0.40, 1.50	NA	NA	1.03	0.35, 3.08	0.84	0.48, 1.49	0.56
**Substantial**	0.72	0.15, 3.57	NA	NA	0.18	0.04, 0.81	0.35	0.09, 1.39	0.14

**Table 7:** Meta-analysis of adverse events or complications

**Complications –no./total no.**	**Closure trial**		**PC trial**		**Respect trial**		**Random effects model**		
	**Device**	**Medical**	**Device**	**Medical**	**Device**	**Medical**	**OR**	**95% CI**	**p-value**
**Major bleeding**	10/378	4/374	7/204	12/210	8/499	9/481	1.02	0.46, 2.27	0.97
**Atrial fibrillation**	23/402	3/458	5/204	2/210	15/499	8/481	3.43	1.17, 10.00	0.02

## Discussion

In this meta-analysis, we analyzed and reported aggregate data for adult patients with PFO and cryptogenic stroke from three large randomized trials comparing standard medical therapy versus transcatheter closure [22-24]. Our study is limited by the heterogeneity of data available from these three clinical trials, such as type of devise used for closure, drugs used for medical therapy, patient population, geographic location within the small number of included trials, particularly with regards the CLOSURE 1 trial. The Starflex closure device employed in the trial, as compared to the Amplatzer device in PC and RESPECT has since been discontinued, partially due to the high device complication rate reported in CLOSURE 1 and association of the device towards the later development of atrial fibrillation (5.7% vs. 0.7%, p < 0.001), thrombosis and mechanical complications, all possible contributor towards recurrent stroke. Similarly, the rate of successful PFO closure was 89.4% in the CLOSURE I trial, as compared to a very high success rate of more than 96% in the PC and RESPECT trials, suggesting superiority of the Amplatzer device compared to the STARFlex device. Furthermore, the definition of neurological event inclusion criteria was not as strict in CLOSURE 1 and did not mandate MRI neuro-radiological evidence of an ischemic event in the setting of TIA. Follow-up was shorter than in PC and RESPECT (2 years vs. 4.1 and 2.6 years), which may have decreased the ability to detect a treatment effect due to the relative rarity of recurrent TIA or strokes. These events may have only been observed with longer follow-up. We report analysis of aggregate data from three RCTs, but meta-analysis of individual patient data may provide clear and better understanding of the benefit of one treatment modality over other. Due to the unavailability of individual patient data, we could not analyze adverse events in detail.

Despite ambiguity regarding patient selection, less than optimum patient enrollment, and inadequate follow up, our meta-analyses which combined data from the three RCTs showed a trend towards superiority of PFO closure as compared to medical therapy, and statistically significant benefit of PFO closure in the per protocol population. Similar results were found when the Amplatzer PFO device was analyzed in the RESPECT trial after accounting for an unequal drop-out rate with per-protocol and as-treated method, which then demonstrated significant benefit with device closure. Likewise, results from the underpowered PC trial (414 patients) were not-statistically significant but the trend for the end-point of stroke and TIA uniformly favored the Amplatzer device.

Case-series and retrospective analyses of patients with PFO and cryptogenic stroke have previously suggested that transcatheter closure of PFO for prevention of recurrent stroke is a highly efficacious and safe procedure [28-32]. Two recent meta-analyses of observational studies showed that transcatheter closure was superior to medical management, with the incidence of recurrent strokes being more than 6 times higher in medically treated patients than those who underwent transcatheter closure [25,26]. The number of patients reported in observational studies were at least 8-fold larger than the total number of patients enrolled in the three RCTs. Similarly, over 15,000 closure procedures have been reported in the literature in observation studies or case series, as compared to only 1,103 patients reported in RCTs. This could be due to rapid adoption of this intervention by many clinical practitioners due to the observation that transcatheter closure is a safe and effective procedure. There are several features about the three randomized trials that are worth considering: all the trials included relatively younger patients with cryptogenic strokes and TIAs. The outcome rate in patients receiving medical therapy was lower compared to published observational studies in the literature, suggesting that the baseline risk in the trial population may have been lower. This may point to a failure to appropriately select patients in the clinical trials who were more likely to have had PFO-related events, possibly attributable to the preference of patients to undergo PFO closure coupled with the reluctance of physicians to randomize patients with true PFO related cryptogenic stroke, such as the ones with clinical indicators of paradoxical embolism (e.g. very large shunt, associated atrial septal aneurysm), or absence of any conventional stroke risk factors. These patients then could have been preferentially treated with off-protocol transcatheter closure. This hypothesis is strongly supported by the findings from a study by the Cleveland Clinic, which indicates that off-label PFO closures out-numbered patient recruitment into the CLOSURE I trial by 3:1 at their institution during the study recruitment period (32). They also found that the large shunts were considerably more common in off-label patients, suggesting that higher-risk patients may have been favorably closed off-label. On the other hand, outcome rates in the closure group, especially in the CLOSURE I trial were higher compared to previous estimates from observational studies.

One can also argue that the benefits of transcatheter closure may become apparent only with longer follow-up, as this intervention is expected to prevent the occurrence of a relatively rare clinical outcome. The 2 to 4 years of follow-up may be inadequate to fully capture the benefit of PFO closure. A study with a long follow-up of up to 15 years, showed that percutaneous PFO closure appeared equally effective for secondary stroke prevention (0.59 (0.26–1.34) p = 0.21) and more effective for secondary TIA prevention (0.19 (0.08–0.49)p = 0.001) compared with medical treatment in patients with PFO [33]. PFO closure studies such as CryptoCard (Trials register #NCT01018355) have been terminated due to dissatisfactory enrollment rate.

It is important to recognize difficulties encountered in enrollment of patients in such trials and the possibility that results from three individual RCTs may make it even more difficult to enroll sufficient patients in the future to detect an event with a very low clinical rate. We await the results of 3 ongoing large randomized trials (REDUCE, CLOSE, and DEFENSE-PFO), which aim to study the relative benefit of anticoagulation, antiplatelet and transcatheter device closure with the Amplatzer and GORE Septal Occluder.

## Conclusions

The results of our meta-analysis show a favorable trend towards transcatheter closure of PFO as compared to medical therapy in intention-to-treat analysis and confirm a benefit in per-protocol analysis, despite the fact that many patients who would have truly benefited from device closure may have not been randomized in these trials, but underwent off-label closure. Device-related complications were higher with the STARFlex device compared to the Amplatzer device, with atrial fibrillation being the most frequent complication. A significant benefit of transcatheter PFO closure was apparent in male patients. Based on current evidence, it is premature to conclude that transcatheter closure of a PFO is futile in cryptogenic stroke, and we therefore advocate continued randomization of future patients in ongoing trials designed to answer the question. Our finding of possible increased selective benefit in males is hypothesis generating and may deserve specific study.

## Abbreviations

PFO: Patent foramen ovale; TIA: Transient ischemic attack; TEE: Transesophageal echocardiography; CLOSURE I: Evaluation of the STARFlex septal closure system in patients with a stroke and/or transient ischemic attack due to presumed paradoxical embolism through a patent foramen ovale; PC: Clinical trial comparing percutaneous closure of patent foramen ovale using the amplatzer PFO occluder with medical treatment in patients with cryptogenic embolism; RESPECT: Randomized evaluation of recurrent stroke comparing PFO closure to established current standard of care treatment.

## Competing interests

No significant competing interests present.

## Authors’ contributions

IR MD: Conception and design, analysis and interpretation of data, drafting of manuscript. AD MD: Conception and design, analysis and interpretation of data, drafting of manuscript, revising critically for important intellectual content. AM MD: Drafting of manuscript. CHH PhD: Conception and design, analysis and interpretation of data. MH MD: Analysis and interpretation of data, drafting of manuscript. JZL MD: Analysis and interpretation of data, drafting of manuscript. KL MD: Revising critically for important intellectual content, final approval of manuscript submitted. KSL MD: Conception and design; analysis and interpretation of data, drafting of manuscript, revising critically for important intellectual content, final approval of manuscript submitted. All authors read and approved the final manuscript.

## Pre-publication history

The pre-publication history for this paper can be accessed here:

http://www.biomedcentral.com/1471-2261/13/116/prepub

## Supplementary Material

Additional file 1 Search Strategy. Click here for file
