# Supplementary material for: Transcatheter patent foramen ovale closure versus medical therapy for cryptogenic stroke: a meta-analysis of randomized clinical trials
Source: BMC Cardiovasc Disord. 2013 Dec 11;13:116. doi: 10.1186/1471-2261-13-116 (PMC3890573; doi:10.1186/1471-2261-13-116)
Supplement: Additional file 1 — Search Strategy. [file 1471-2261-13-116-S1.pdf]

Search Strategy

Clinical question type : Intervention

1. Stroke/
2. "Intracranial Embolism and Thrombosis"/
3. (crypt$ adj25 stroke).tw.
4. 1 or 2 or 3
5. Foramen Ovale, Patent/
6. 4 and 5
7. (device adj10 closure).tw.
8. transcath$.tw.
9. closur*.tw.
10. 7 or 8 or 9
11. medic*.tw.
12. 6 and 10 and 11
13. review.pt. and medline.tw.
14. meta analysis.pt.
15. (systematic$ and (review* or overview*)).tw.
16. meta?analy$.tw.
17. meta analy$.tw.
18. or/13-17
19. randominzed controlled trial.pt.
20. vcontrolled clinical trial.pt.
21. randomized.ab.
22. placebo.ab.
23. clinical trial as topic/
24. randomly.ab.
25. trial.ti.
26. or/19-25
27. 12 and 18
28. 12 and 26
